# Diagnostic performance of regional systematic biopsy for prostate cancer stratified by PI-RADS and histologic zones

**DOI:** 10.1186/s13244-024-01680-1

**Published:** 2024-05-16

**Authors:** Zhoujie Sun, Yi Liu, Wei Yu, He Wang, Qi Shen, Cuijian Zhang

**Affiliations:** 1 Department of Urology, Peking University First Hospital, No.8 Xishiku St. Xicheng District, Beijing, China; 2 Institute of Urology, Peking University, No.8 Xishiku St. Xicheng District, Beijing, China; 3 The National Urological Cancer Center of China, No.8 Xishiku St. Xicheng District, Beijing, China; 4 Department of Radiology, Peking University First Hospital, Beijing, 100034 China

**Keywords:** Image-guided biopsy, Prostatic neoplasms, Magnetic resonance imaging

## Abstract

**Objectives:**

To explore the diagnostic performance of targeted biopsy (TB) combined with regional systematic biopsy (RSB) in patients with different Prostate Imaging Reporting and Data System (PI-RADS) and histologic zones for prostate lesions.

**Methods:**

This retrospective study included 1301 patients who underwent multiparametric MRI followed by combined MRI/US fusion-guided TB+systematic biopsy (SB) between January 2019 and October 2022. RSB was defined as the four perilesional SB cores adjacent to an MRI-positive lesion. Cancer detection rates were calculated for TB + SB, TB, SB, and TB + RSB, while the McNemar test was utilized for multiple comparisons among them. Subgroup analyses were performed based on different Pl-RADS and histologic zones.

**Results:**

Of 1301 included participants (median age, 68 years; interquartile range, 63–74 years), 16,104 total biopsy cores were performed. TB + RSB detected clinically significant prostate cancer in 70.9% (922/1301) of patients, which was significantly higher than TB (67.4%, *p* < 0.001) or SB (67.5%, *p* < 0.001) but similar to TB + SB (71.0%, *p* = 0.50). Compared with TB + SB, TB + RSB required fewer median biopsy cores (6.3 vs. 12.4, *p* < 0.001) and had a higher proportion of positive cores (56.3% vs. 39.0%, *p* < 0.001). Subgroup analysis showed that TB had outstanding sensitivity for detecting PI-RADS 5 lesions in the PZ.

**Conclusions:**

Compared with TB + SB, TB + RSB achieved a similar clinically significant prostate cancer detection rate while requiring fewer biopsy cores and exhibiting higher diagnostic efficiency.

**Critical relevance statement:**

For MRI-positive prostate lesions, targeted biopsy combined with regional systematic biopsy could serve as an alternative diagnostic approach to targeted biopsy combined with systematic biopsy.

**Key Points:**

- The scheme of prostate biopsy needs to be optimized.
- Regional systematic biopsy decreases the total number of cores taken.
- Targeted biopsies combined with regional systematic biopsies improve prostate diagnostic efficiency.

**Graphical Abstract:**

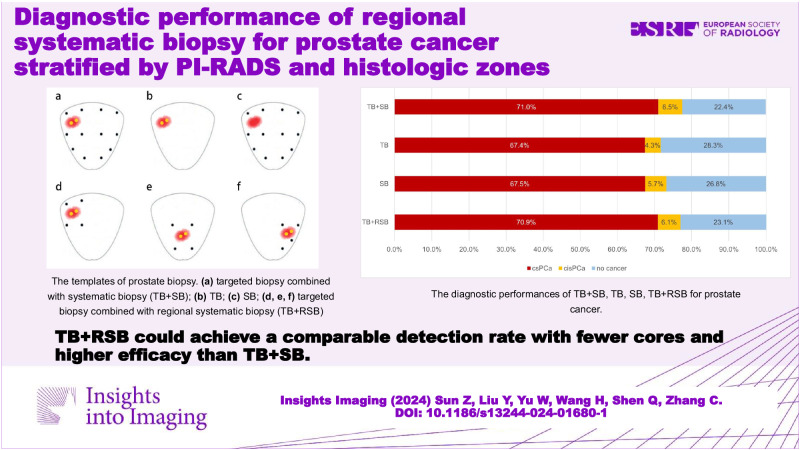

## Introduction

The European Association of Urology (EAU) guidelines in 2022 recommended performing combined multiparametric magnetic resonance imaging (mpMRI) targeted biopsy (TB) and ultrasound-guided systematic biopsy (SB) in biopsy-naïve patients with positive MRI findings [1]. However, the combined biopsy (TB + SB) approach requires more biopsy cores than either TB alone or SB alone, potentially leading to increased complications and intraoperative discomfort. Furthermore, most patients benefit diagnostically from TB cores rather than SB, as SB detects higher rates of clinically insignificant cancer (cisPCa) [2–7]. Therefore, it is necessary to explore the sampling schemes to minimize biopsy cores while maximize cancer detection.

Given the higher efficacy of TB in the diagnosis of clinically significant prostate cancer (csPCa), the research on optimizing the prostate sampling schemes was mainly focused on retaining TB cores and reducing the number of SB cores. Several studies have explored sampling schemes reducing the ipsilateral or contralateral SB cores [8, 9]. However, these studies ignored the categorization of Prostate Imaging Reporting and Data System (PI-RADS) or histologic zones of MRI lesion.

TB combined with regional systematic biopsies (RSB) rather than standard SB have been explored as an alternative strategy to minimize biopsy cores. Some articles and meta-analysis have reported similar csPCa detection rates for TB + SB and TB + RSB [10, 11]. Nevertheless, these approaches did not significantly reduce the number of biopsy cores.

As PI-RADS v2.1 recommended, lesions in the peripheral zone (PZ) and transitional zone (TZ) are assessed by different scoring principles [12]. Therefore, it is necessary to stratify the MRI suspicious lesions based on different PI-RADS and histologic zones and individualize the sampling schemes reducing the biopsy cores for patients.

Currently, there is no consensus on the specific definition for RSB. Several RSB templates can be found in literatures, including four perilesional systematic cores, cores from adjacent sectors, two adjacent sector cores within a 2-cm of MRI lesions cores, and so on [11, 13–15]. Large prospective multicenter research has found that the addition of four perilesional cores improved the detection of csPCa [14]. This demonstrates that TB + RSB (four perilesional systematic cores) that adequately sampling the tissue adjacent to MRI lesion tissue could achieve a comparable detection rate with fewer cores.

The primary endpoint of our study was the cancer detection rate and diagnostic performances of TB + SB, TB + RSB, TB, or SB in MRI-positive patients. More importantly, we analyzed and individualized the sampling schemes of patients for different PI-RADS and histologic zones as subgroups within this cohort.

## Materials and methods

### Ethics approval and consent

The study was performed in accordance with the ethical standards as laid down in the 1964 Declaration of Helsinki and its later amendments or comparable ethical standards and approved by the Ethics Committee of Peking University First Hospital (protocol code 2016–1252). Informed consent with guarantees of confidentiality was provided from all human subjects involved in the study. It was a retrospective observational study.

### Patients selection

**Fig. 1:**
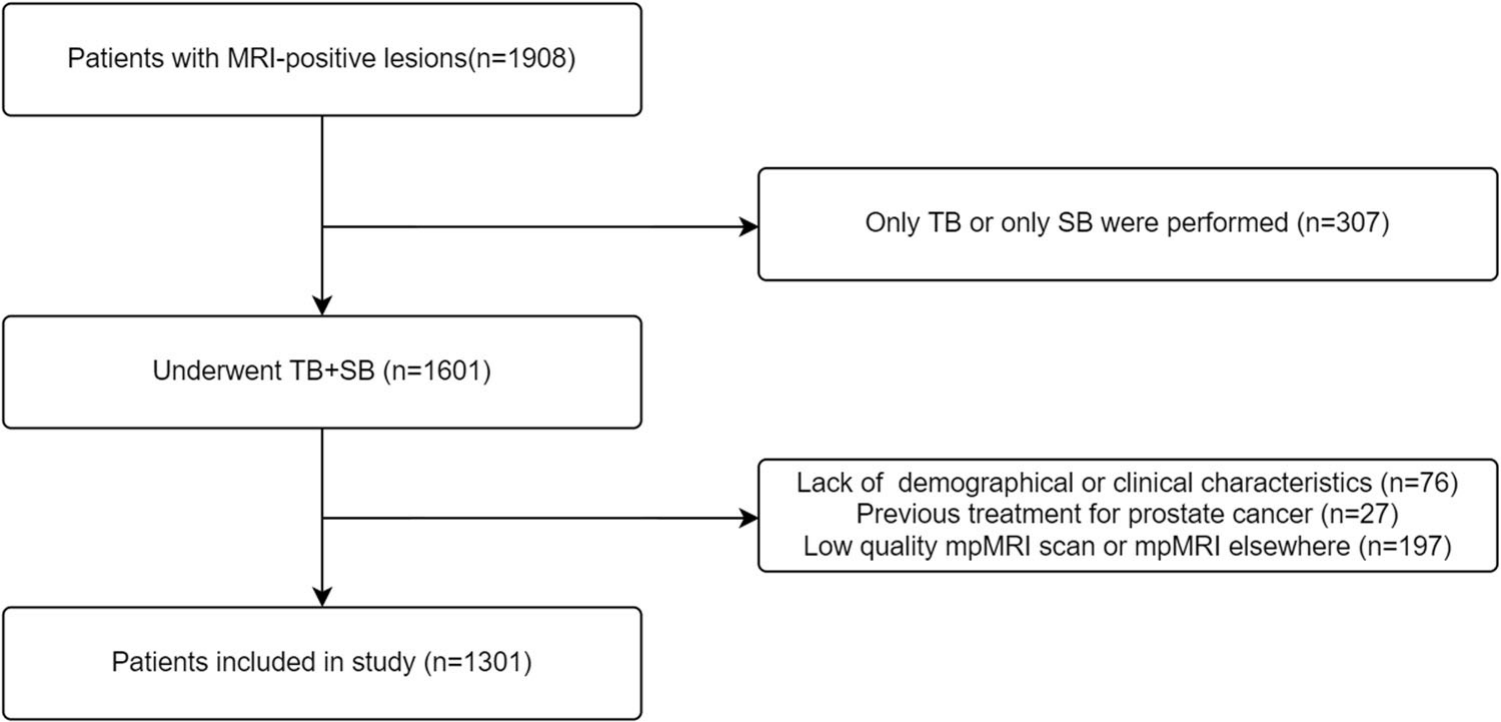
Flow diagram of the patients included in the study

From January 2019 to October 2022, a total of 1908 men with at least one MRI-positive lesion, defined as PI-RADS ≥ 3, were retrospectively recruited. 1601 patients underwent TB + SB as their naïve-biopsy in the Department of Urology at Peking University First Hospital. These patients received at least 2 core TB and 8 core SB, which coincided with recommendations from EAU guidelines and others [1]. The exclusion criteria were lack of demographical or clinical characteristics (*n* = 76), previous treatment for prostate cancer (*n* = 27), low-quality mpMRI scan and mpMRI elsewhere (*n* = 197). Finally, 1301 patients undergoing 16,104 total cores were enrolled in the study (Fig. 1).

### mpMRI examination

Men enrolled underwent mpMRI due to the clinical suspicion of prostate cancer for elevated serum total prostate-specific antigen (tPSA) level or/and an abnormal digital rectal examination. They were conducted by a 3.0 T Discovery MR750 HDx (GE Healthcare, Waukesha, WI, USA) with a phased-array coil. The imaging protocol included T1-weighted imaging of the pelvis, T2-weighted imaging fast spin-echo images centered on the prostate, diffusion-weighted imaging and corresponding apparent diffusion coefficient maps, and dynamic contrast-enhanced images. The mpMRI protocol satisfied the recommended minimum standards set by consensus guidelines [16].

### Biopsy procedure and histopathology

**Fig. 2:**
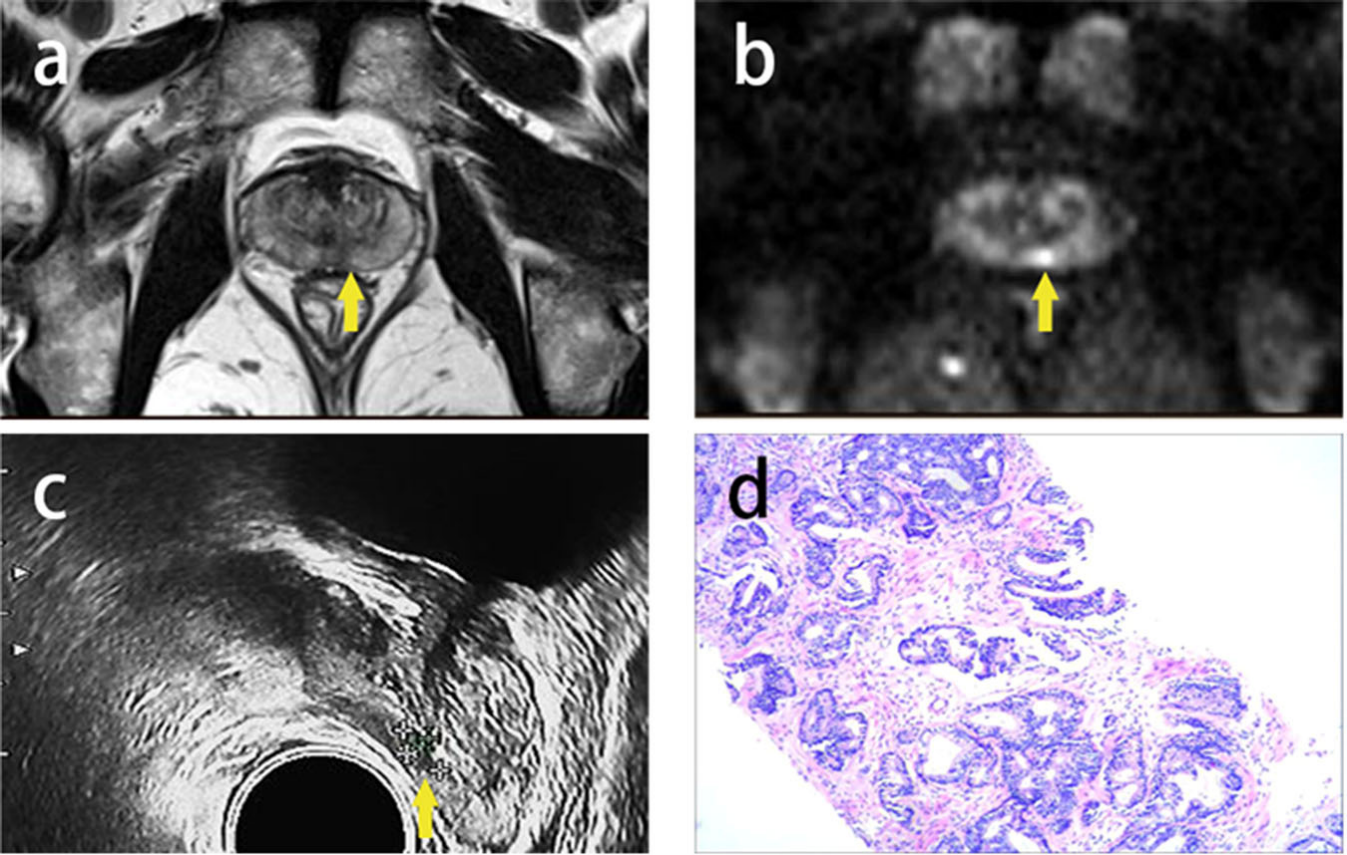
Imaging in a 67-year-old man with prostate-specific antigen 8.8 ng/mL. The suspected PI-RADS 3 lesion was in the left PZ of midgland. **a** Mildly hypointensity on T2WI (arrow); **b** Mildly hyperintense on DWI (arrow); **c** Hypoechoic on transrectal ultrasound (arrow); **d** Prostate adenocarcinoma, Gleason Score 4 + 3 (SUM = 7). T2WI, T2-weighted imaging; DWI, diffusion-weighted imaging

All patients enrolled underwent transrectal ultrasound-guided 8–12 cores freehand SB followed by 2–5 TB. The TB was conducted using cognitive MRI/US fusion. All biopsies were undertaken by three urologists with > 10 years of experience. All biopsy specimens were evaluated by a dedicated genitourinary pathologist with > 10 years of experience. The specimens were reported according to the recommendations of the International Society of Urological Pathology [17]. The csPCa was defined as International Society of Urological Pathology grade ≥ 2 [18]. Figure 2 shows the examination results of one patient, including mpMRI, biopsy procedure, and histopathology.

### The definition and calculation of RSB

**Fig. 3:**
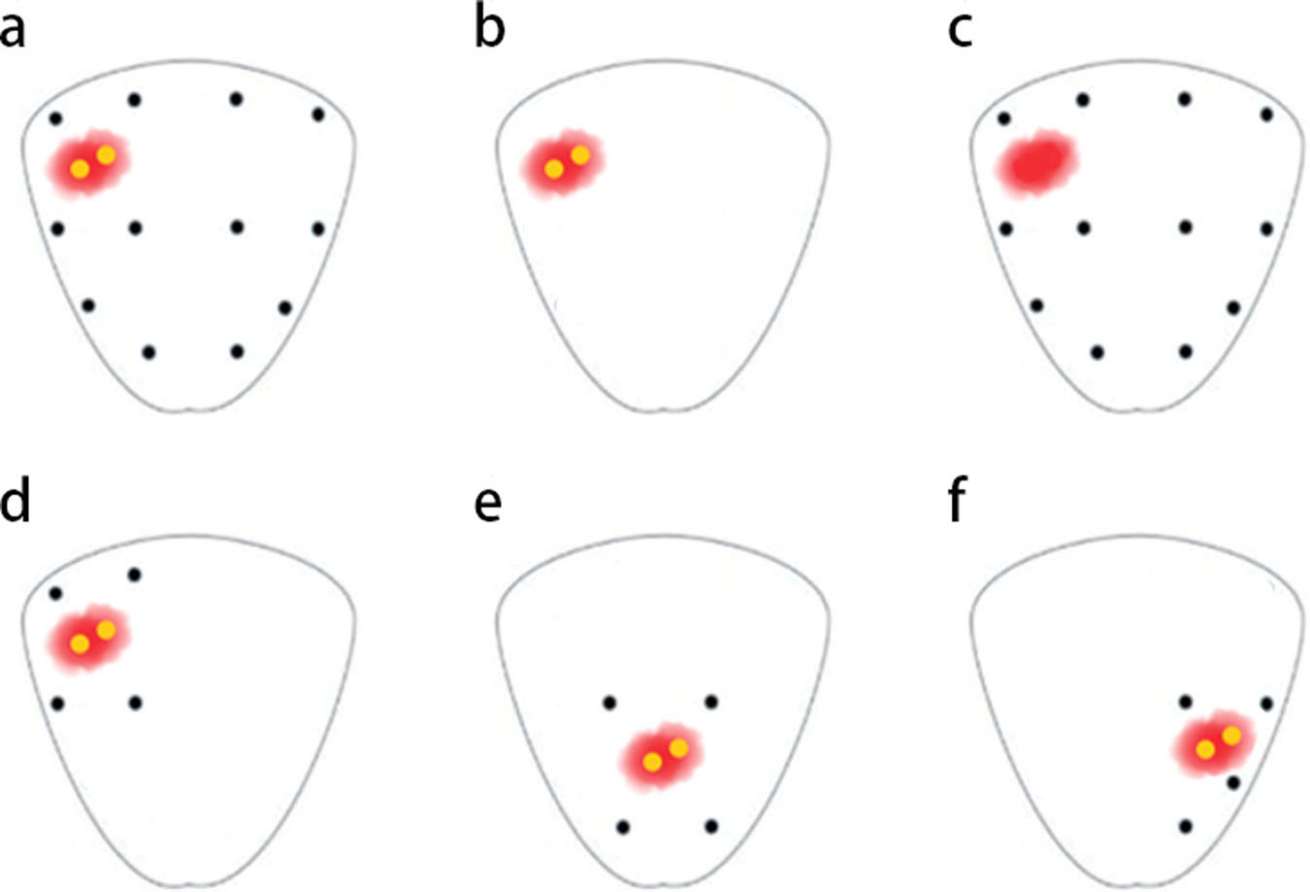
The templates of prostate biopsy. The red region shows the location of the suspected lesion. Yellow dots show the targeted biopsy core, and black dots show the systematic biopsy core; **a** targeted biopsy combined with systematic biopsy (TB + SB); **b** targeted biopsy (TB); **c** systematic biopsy (SB); **d** targeted biopsy combined with regional systematic biopsy (TB + RSB); **e**, **f** TB + RSB for a lesion at other location

It was a self-controlled design for different biopsy approaches. The RSB was defined as the four perilesional transrectal systematic biopsy cores adjacent to the MRI-positive lesion. Different biopsy templates are shown in Fig. 3.

### Statistical methods

Continuous variables were described as medians with interquartile ranges (IQRs), and categorical variables were reported as proportions and percentages for patients’ characteristics. The McNemar test was used for multiple comparisons between TB + SB, TB, SB, and TB + RSB, and the Kappa test was used to evaluate the consistency at the same time. Different PI-RADS and histologic zones were divided as the subgroups of the cohort. Patients were classified into groups of no cancer, cisPCa and csPCa to describe baseline clinical and pathological characteristics, and continuous variables were compared using the Kruskal-Wallis test. The Receiver Operating Characteristic (ROC) curves for PI-RADS, prostate-specific antigen density (PSAD), tPSA, and maximum Diameter were plotted in predicting prostate cancer (PCa) and csPCa. Using the TB + SB standard, we analyzed the sensitivity of TB, SB, and TB + RSB for PCa or csPCa detection rates, respectively. All tests were two-sided, and statistical significance was set at *p* < 0.05. Statistical analyses were performed by SPSS (version 24.0; SPSS Inc., Chicago, IL, USA).

## Results

### Patient characteristics

A total of 1301 patients with a median PSA of 12.3 ng/mL (IQR 7.5, 27.1) were enrolled in the study (Fig. 1). Baseline clinical and pathological characteristics were described in Table 1. Of the 304 (23.4%) patients were PI-RADS 3, 31.7% PI-RADS 4, and 45.0% PI-RADS 5. In all, 1009(77.6%) men had PCa, 924 (71.0%) of whom had csPCa.

**Table 1:** Baseline clinical and pathological characteristics of the patients

	Total (*n* = 1301)	No cancer (*n* = 292)	cisPCa (*n* = 85)	csPCa (*n* = 924)
**Median (IQR)**				
Age (year)	68 (63–74)	64 (59–69)	69 (64–73)	70 (64–76)
tPSA (ng/mL)	12.3 (7.5–27.1)	8.5 (6.1–12.9)	8.7 (6.1–11.5)	15.6 (8.9–38.8)
PV (mL)	49 (35–72)	66 (46–90)	47 (31–68)	46 (33–64)
PSAD (ng/mL^2^)	0.27 (0.14–0.60)	0.13 (0.09–0.19)	0.17 (0.12–0.30)	0.38 (0.20–0.85)
D (cm)	1.6 (1.1–2.5)	1.2 (0.9–1.6)	1.2 (0.8–1.7)	1.9 (1.3–3.0)
***n*** **(%)**				
PI-RADS				
3	304 (23.4)	190 (65.1)	33 (38.8)	81 (8.5)
4	412 (31.7)	77 (26.4)	41 (48.2)	294 (30.9)
5	585 (45.0)	25 (8.6)	11 (12.9)	549 (57.8)
Histologic zones				
PZ	988 (75.9)	167 (57.2)	43 (50.6)	778 (84.2)
TZ	727 (55.9)	150 (51.3)	51 (60.0)	526 (56.9)
AS	363 (27.9)	23 (7.8)	21 (24.7)	319 (31.6)
CZ	226 (17.4)	23 (7.8)	0 (0)	203 (20.1)
T				
T2a	576 (44.3)	182 (62.3)	55 (64.7)	339 (36.7)
T2b	41 (3.2)	7 (2.4)	4 (4.7)	30 (3.2)
T2c	332 (25.5)	94 (32.2)	24 (28.2)	214 (23.2)
T3a	75 (5.8)	3 (1.0)	1 (1.2)	71 (7.6)
T3b	139 (10.7)	4 (1.4)	0 (0)	135 (14.6)
T4	138 (10.6)	2 (0.6)	1 (1.2)	135 (14.6)

*PV* prostate volume, *D* maximum diameter, *AS* anterior fibrous muscle matrix, *CZ* central zone

**Fig. 4:**
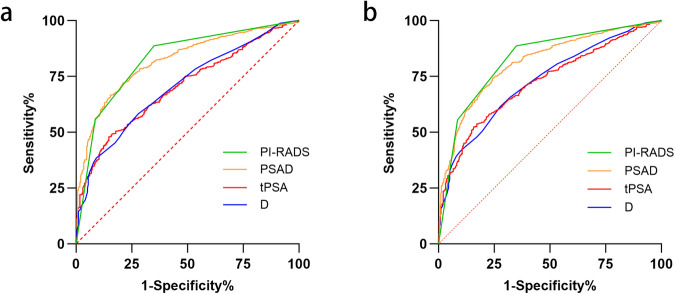
Roles of PI-RADS, PSAD, tPSA, and maximum Diameter in predicting prostate cancer (**a**) and clinically significant prostate cancer (**b**) by ROC analysis

The ROC curve for PI-RADS, PSAD, tPSA, and maximum diameter was plotted in predicting PCa and csPCa (Fig. 4). For PCa, the area under curve (AUC) of PI-RADS, PSAD, tPSA, and maximum diameter was 0.828 (95%CI: 0.801–0.855, *p* < 0.001), 0.819 (95%CI: 0.794–0.844, *p* < 0.001), 0.702 (95%CI: 0.671–0.732, *p* < 0.001), and 0.704 (95%CI: 0.673–0.736, *p* < 0.001), respectively. For csPCa, the AUC of PI-RADS, PSAD, tPSA, and maximum diameter was 0.830 (95%CI: 0.805–0.855, *p* < 0.001), 0.812 (95%CI: 0.788–0.837, *p* < 0.001), 0.723 (95%CI: 0.695–0.752, *p* < 0.001), and 0.728 (95%CI: 0.700–0.757, *p* < 0.001).

### The PCa detection rate of different prostate sampling schemes

**Fig. 5:**
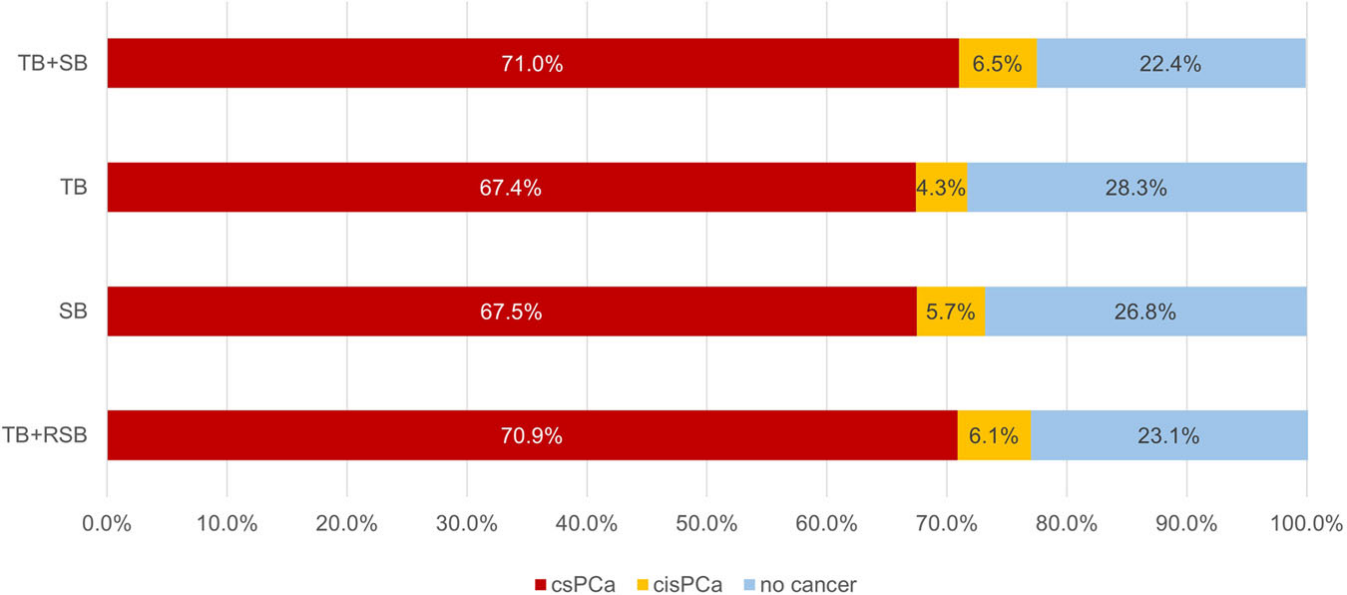
Percentages of men with csPCa, cisPCa, and no cancer with different prostate sampling scheme

The cancer detection rates of different prostate sampling schemes are shown in Fig. 5, and Kappa values for the consistency were provided in Table 2. TB + SB, TB, SB, TB + RSB detected csPCa in 71.0% (924/1301), 67.4% (877/1301), 67.5% (878/1301), and 70.9% (922/1301) of patients, respectively.

**Table 2:** Diagnostic performances of different prostate sampling schemes compared with TB + SB

	n	PCa	*p*^a^	Kappa^a^	csPCa	*p*^a^	Kappa^a^
TB + SB	1301	1009 (77.6)	–	–	924 (71.0)	–	–
TB	1301	933 (71.7)	< 0.001	0.846	877 (67.4)	< 0.001	0.915
SB	1301	952 (73.2)	< 0.001	0.882	878 (67.5)	< 0.001	0.917
TB + RSB	1301	1001 (76.9)	0.008	0.983	922 (70.9)	0.50	0.996

^a^ Compared with TB + SB

Although TB + SB had a higher detection rate of PCa compared to TB + RSB (77.6% vs. 76.9%, *p* = 0.008), they did not significantly differ in overall csPCa detection (71.0% vs. 70.9%, *p* = 0.50). However, combined biopsy detected significantly more patients with PCa or csPCa compared to TB or SB. And TB + RSB was significantly higher than the detection by TB (70.9% vs. 67.4%, *p* < 0.001) and SB (70.9% vs. 67.5%, *p* < 0.001) for csPCa (Table 3). TB and SB had similar csPCa detection rates (67.4% vs. 67.5%, *p* = 1.00), but TB detected fewer cisPCa (4.3% vs. 5.7%, *p* = 0.006).

**Table 3:** Diagnostic performances of different prostate sampling schemes compared with TB + RSB

	*n*	PCa	*p*^a^	Kappa^a^	csPCa	*p*^a^	Kappa^a^
TB + RSB	1301	1001 (76.9)	–	–	922 (70.9)	–	–
TB	1301	933 (71.7)	< 0.001	0.864	877 (67.4)	< 0.001	0.919
SB	1301	952 (73.2)	< 0.001	0.867	878 (67.5)	< 0.001	0.914

^a^ Compared with TB + RSB

### The mean cores and positive cores for different sampling schemes

**Fig. 6:**
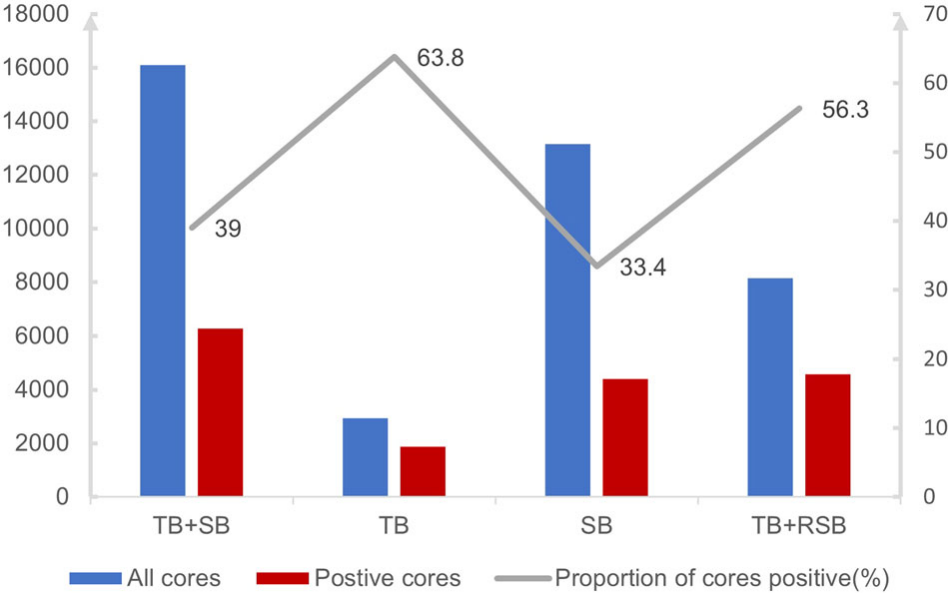
The cores and positive cores for different sampling schemes

TB + SB utilized an average of 12.4 cores, while TB + RSB utilized an average of 6.3 cores per MRI-positive lesion (Table 4 and Fig. 6). The TB + RSB approach resulted in a 49.4% decrease (7958/16,104) in the overall number of biopsy cores (an average of 6.1 cores per patient) when compared to combined biopsy. Meanwhile, SB utilized an average of 10.1 cores with the lowest proportion of positive cores. Compared with TB + SB, TB + RSB had fewer cores on average (6.3 vs. 12.4, *p* < 0.001) but had a higher proportion of positive cores (56.3% vs. 39.0%, *p* < 0.001).

**Table 4:** The cores and positive cores for different sampling schemes

	TB + SB	TB	SB	TB + RSB
All cores	16,104	2942	13,162	8146
Positive cores	6276	1877	4399	4584
Proportion of positive cores (%)	39.0	63.8	33.4	56.3
Mean ± SD cores	12.4 ± 3.0	2.3 ± 0.9	10.1 ± 2.3	6.3 ± 0.9
Mean ± SD positive cores	4.8 ± 3.7	1.4 ± 1.1	3.4 ± 3.0	3.5 ± 2.4

*SD* Standard Deviation

### Diagnostic performances of different sampling schemes in PZ or TZ

For patients with lesions in PZ, TB + SB and TB + RSB had similar detection rates for PI-RADS 3-5 whatever about PCa or csPCa (Table 5). Of note, although TB + SB had higher PCa or csPCa detection rates compared to TB for PI-RADS 3-4 lesions in PZ, they did not significantly differ in PI-RADS 5.

**Table 5:** Diagnostic performances of different prostate sampling schemes in PZ

PI-RADS		*n*	PCa	*p*^a^	Kappa^a^	csPCa	*p*^a^	Kappa^a^
3	TB + SB	158	64 (40.5)	–	–	52 (32.9)	–	–
	TB	158	51 (32.3)	< 0.001	0.824	43 (27.2)	0.004	0.865
	SB	158	61 (38.6)	0.25	0.96	49 (31.0)	0.25	0.956
	TB + RSB	158	63 (39.9)	1	0.987	51 (32.3)	1	0.986
4	TB + SB	325	271 (83.4)	–	–	243 (74.8)	–	–
	TB	325	234 (72.0)	< 0.001	0.678	218 (67.0)	< 0.001	0.815
	SB	325	255 (78.5)	< 0.001	0.841	228 (70.1)	< 0.001	0.855
	TB + RSB	325	269 (82.8)	0.50	0.978	243 (74.8)	1	1
5	TB + SB	505	486 (96.2)	–	–	483 (95.6)	–	–
	TB	505	482 (95.4)	0.125	0.901	479 (94.9)	0.125	0.913
	SB	505	484 (95.8)	0.50	0.948	481 (95.2)	0.50	0.954
	TB + RSB	505	486 (96.2)	1	1	483 (95.6)	1	1

^a^ Compared with TB + SB

For patients with lesions in TZ, TB + RSB also achieved a comparable detection rate with TB + SB in PI-RADS 3-5 (Supplementary Table S1). Being different from lesions in PZ, TB + SB detected significantly more patients than TB for TZ lesions.

## Discussion

The traditional TB + SB performed excess biopsy cores resulting in more complications and intraoperative discomfort. TB + RSB were explored as an alternative strategy to minimize biopsy cores. In the present study, we focused on the diagnostic performance of TB + RSB. Consistent with previous research, we found that TB + RSB detected significantly more csPCa than SB or TB and was nearly equivalent to TB + SB (70.9% vs. 71.0%, *p* = 0.50, Kappa = 0.996). TB + RSB could achieve a comparable detection rate, while requiring fewer biopsy cores and exhibiting higher diagnostic efficacy.

Several patients included in the study had more than one lesion. Multicenter research, however, has shown that the main lesion played a major role in patients with multiple lesions, and TB of secondary lesions could be safely omitted [19]. The patients enrolled in our research showed the analogous results. None of the patients with multiple lesions had positive secondary lesions and negative primary lesions. Thus, the RSB (four perilesional systematic cores) were taken around the main MRI-positive lesions in the study.

Using combined biopsy as the reference standard, TB, SB, and TB + RSB detected 92.5% (933/1009), 94.4% (952/1009), 99.2% (1001/1009) PCa and 94.9% (877/924), 95.0% (878/924), 99.8% (922/924) csPCa, respectively. Only two cases of csPCa were missed by TB + RSB, which was superior to TB or SB (0.2% vs. 5.1%, *p* < 0.001; 0.2% vs. 5.0%, *p* < 0.001). This result tied well with previous studies [10, 13, 14]. The TB + 4 perilesional SB approach in our cohort almost detected all cases of csPCa and reduced 6.1 biopsy cores on average.

PI-RADS and histologic zones were specifically discussed in a relatively large number of patients, which was a major advantage of our research. PI-RADS standardized the reports of prostate mpMRI and had distinguished value of clinical application [20]. Several studies have suggested that PI-RADS was an independent predictor for csPCa diagnosis [21]. Our research also demonstrated that PI-RADS was a well-performing predictor for csPCa (AUC: 0.830, 95%CI: 0.805–0.855, *p* < 0.001). As PI-RADS v2.1 recommended, lesions in the PZ were mainly scored by diffusion-weighted imaging and adjusted by dynamic contrast-enhanced. Lesions in the TZ were assessed by T2-weighted imaging and adjusted by diffusion-weighted imaging [12]. Due to the distinct scoring principles for PZ and TZ, it was essential to classify the PI-RADS and histologic zones when thoroughly analyzing detection rates.

For patients with lesions in PZ, TB + SB and TB + RSB had comparable detection rates for all PI-RADS. However, the remarkable finding was that TB also had a surprising detection rate for PI-RADS 5 in PZ lesion whatever PCa or csPCa (95.4% vs. 96.2%, *p* = 0.125; 94.9% vs. 95.6%, *p* = 0.125, respectively). Only four (0.8%) csPCa cases were missed by TB in these patients. Indeed, in most cases, TB + RSB offered a significant advantage over TB alone. However, this advantage is not apparent in PI-RADS 5 in the PZ (Supplementary Tables S2, 3). SB also showed the same results for PI-RADS 5 PZ lesion, but it required a significantly higher number of cores, resulting in lower efficiency. For TZ PI-RADS 5 lesion, however, TB alone was insufficient to diagnosis due to its inferior detection rates than TB + SB. Above all, for lesions in TZ or PI-RADS 3-4 lesions in PZ, TB + RSB could be the practicable substitute for TB + SB. For PI-RADS 5 lesions in PZ, just TB alone might be sufficient to diagnosis. Of course, further prospective multicenter studies were required to validate this approach.

Compared with traditional SB, TB guided by mpMRI has shown a decrease in biopsy cores and detection rates of cisPCa, while increasing the csPCa. Despite the favorable results of TB only, there were also disadvantages such as targeting error and influence by operator experience. SB could make up for the inaccuracy of targeted puncture. Our study showed that the added value of SB for PCa and csPCa detection was 76/1009 (7.5%) and 47/924 (5.1%), respectively. It could be seen that TB and SB had complementary effects. We also found that TB detected fewer cisPCa (4.3% vs. 5.7%, *p* = 0.006), which was in accordance with the previous study [4, 14, 22]. It had been certified that TB + SB could improve csPCa detection and reduce grade misclassification, which was suggested and standardized by EAU guidelines [1].

However, the combined biopsy should not be a simple “combination” of TB and SB, but should be effectively integrating them to optimize the core sampling site and improve the diagnostic efficiency of biopsy for csPCa. TB + RSB had a higher proportion of positive cores and higher diagnostic efficacy with fewer biopsy core numbers, which was expected to reduce complications and patient discomfort during the biopsy.

There were several limitations in our research. First, compared with the literature previous, our proportion of csPCa cases in MRI-positive lesion was significantly higher [4, 6, 23]. This was probably due to the fact that many patients enrolled may have been diagnosed at local hospitals, and our hospital usually treated cases with more complex conditions as a referral center. However, this actually resulted in increasing statistical bias. Second, it was unclear whether these patients underwent prostatectomy in the future. The pathology of combined biopsy may not be surely accurate due to the lack of final surgical pathological results. Finally, our study was a retrospective, single-center study, further prospective multicenter studies were required to validate our novel sampling scheme.

In conclusion, our study demonstrated that TB + RSB detected significantly more csPCa than SB or TB and similar to TB + SB. Furthermore, TB + RSB could achieve a comparable detection rate with fewer cores and higher efficacy. TB and SB had similar csPCa detection rates, but TB detected fewer cisPCa substantially. For lesions in TZ or PI-RADS, 3–4 lesions in PZ, TB + RSB could serve as an alternative diagnostic approach to the TB + SB. For PI-RADS 5 lesions in PZ, employing TB alone may be a compelling diagnostic strategy.

### Material availability

The data that support the findings of this study are available on request from the corresponding author upon reasonable request.

### Supplementary information

Supplementary Information

## Data Availability

The data that support the findings of this study are available on request from the corresponding author upon reasonable request.

## Abbreviations

AUC: Area under curve
cisPCa: Clinically insignificant cancer
csPCa: Clinically significant prostate cancer
EAU: The European Association of Urology
IQR: Interquartile range
mpMRI: Multiparametric magnetic resonance imaging
PCa: Prostate cancer
PI-RADS: Prostate Imaging Reporting and Data System
PSAD: Prostate-specific antigen density
PZ: Peripheral zone
ROC: Receiver Operating Characteristic
RSB: Regional systematic biopsy
SB: Systematic biopsy
TB: Targeted biopsy
tPSA: Total prostate-specific antigen
TZ: Transitional zone
