# Supplementary material for: Diagnostic performance of regional systematic biopsy for prostate cancer stratified by PI-RADS and histologic zones
Source: Insights Imaging. 2024 May 16;15:118. doi: 10.1186/s13244-024-01680-1 (PMC11098978; doi:10.1186/s13244-024-01680-1)
Supplement: Supplementary file 1 — Supplementary Information [file 13244_2024_1680_MOESM1_ESM.pdf]

Diagnostic Performance of Regional Systematic Biopsy for Prostate Cancer Stratified by PI-RADS and Histologic Zones

ELECTRONIC SUPPLEMENTARY MATERIAL

Supplementary Table S1. Diagnostic performances of different prostate sampling schemes in TZ

PI-RADS		n	PCa	*p**	Kappa*	csPCa	*p**	Kappa*
3	TB+SB	167	61 (36.5)	-	-	38 (22.8)	-	-
	TB	167	46 (27.5)	< 0.001	0.796	33 (19.8)	0.063	0.911
	SB	167	52 (31.1)	0.004	0.880	34 (20.4)	0.125	0.929
	TB+RSB	167	58 (34.7)	0.25	0.961	38 (22.8)	1	1
4	TB+SB	141	112 (79.4)	-	-	95 (67.4)	-	-
	TB	141	99 (70.2)	< 0.001	0.758	87 (61.7)	0.008	0.876
	SB	141	95 (67.4)	< 0.001	0.697	80 (56.7)	< 0.001	0.777
	TB+RSB	141	110 (78.0)	1	0.979	95 (67.4)	1	1
5	TB+SB	419	404 (96.4)	-	-	393 (93.8)	-	-
	TB	419	397 (94.7)	0.016	0.802	388 (92.6)	0.063	0.906
	SB	419	393 (93.8)	0.001	0.719	384 (91.6)	0.004	0.841
	TB+RSB	419	404 (96.4)	1	1	393 (93.8)	1	1

*Compared with TB+SB

TB = targeted biopsy; SB = systematic biopsy; RSB = regional systematic biopsy; mpMRI = multiparametric magnetic resonance imaging; PI-RADS = Prostate Imaging

Reporting and Data System; PCa = prostate cancer; csPCa = clinically significant prostate cancer; TZ = transitional zone

Supplementary Table S2. Diagnostic performances of different prostate sampling schemes compared with TB+RSB in PZ

PI-RADS		n	PCa	*p**	Kappa*	csPCa	*p**	Kappa*
3	TB+RSB	158	63 (39.9)	-	-	51 (32.3)	-	-
	TB	158	51 (32.3)	< 0.001	0.836	43 (27.2)	0.008	0.879
	SB	158	61 (38.6)	0.625	0.947	49 (31.0)	0.625	0.941
4	TB+RSB	325	269 (82.8)	-	-	243 (74.8)	-	-
	TB	325	234 (72.0)	< 0.001	0.697	218 (67.0)	< 0.001	0.815
	SB	325	255 (78.5)	< 0.001	0.823	228 (70.1)	< 0.001	0.885
5	TB+RSB	505	486 (96.2)	-	-	483 (95.6)	-	-
	TB	505	482 (95.4)	0.125	0.901	479 (94.9)	0.125	0.913
	SB	505	484 (95.8)	0.5	0.948	481 (95.2)	0.5	0.954

*Compared with TB+RSB

TB = targeted biopsy; SB = systematic biopsy; RSB = regional systematic biopsy; PI-RADS = Prostate Imaging Reporting and Data System; PCa = prostate cancer; csPCa = clinically significant prostate cancer; PZ = peripheral zone

Supplementary Table S3. Diagnostic performances of different prostate sampling schemes compared with TB+RSB in TZ

PI-RADS		n	PCa	*p**	Kappa*	csPCa	*p**	Kappa*
3	TB+RSB	167	58 (34.7)	-	-	38 (22.8)	-	-
	TB	167	46 (27.5)	< 0.001	0.833	33 (19.8)	0.063	0.911
	SB	167	52 (31.1)	0.146	0.838	34 (20.4)	0.125	0.929
4	TB+RSB	141	110 (78.0)	-	-	95 (67.4)	-	-
	TB	141	99 (70.2)	< 0.001	0.778	87 (61.7)	0.008	0.876
	SB	141	95 (67.4)	< 0.001	0.681	80 (56.7)	< 0.001	0.777
5	TB+RSB	419	404 (96.4)	-	-	393 (93.8)	-	-
	TB	419	397 (94.7)	0.016	0.802	388 (92.6)	0.063	0.906
	SB	419	393 (93.8)	< 0.001	0.719	384 (91.6)	0.004	0.841

*Compared with TB+RSB

TB = targeted biopsy; SB = systematic biopsy; RSB = regional systematic biopsy; PI-RADS = Prostate Imaging Reporting and Data System; PCa = prostate cancer; csPCa = clinically significant prostate cancer; TZ = transitional zone
